# Synergism in Two-Component Insecticides with Dillapiole against Fall Armyworm

**DOI:** 10.3390/plants12173042

**Published:** 2023-08-24

**Authors:** Murilo Fazolin, Humberto R. Bizzo, André F. M. Monteiro, Maria E. C. Lima, Natália S. Maisforte, Paola E. Gama

**Affiliations:** 1 Embrapa Acre, Rodovia BR 364, km 14, Rio Branco 69900-970, AC, Brazil; andre.monteiro@embrapa.br; 2 Embrapa Agroindústria de Alimentos, Avenida das Américas 29501, Rio de Janeiro 23020-470, RJ, Brazil; paola.gama@embrapa.br; 3 Programa de Pós-Graduação em Agropecuária nos Trópicos Úmidos, Universidade Federal do Acre, Rodovia BR 364, km 04, Rio Branco 69920-900, AC, Brazil; erica231197@gmail.com; 4 Instituto Federal do Acre, Avenida Brasil 920, Rio Branco 69903-068, AC, Brazil; nataliamaisforte200@gmail.com

**Keywords:** essential oils, insecticidal activity, *Piper aduncum*, *Spodoptera frugiperda*, synergism

## Abstract

The fall armyworm (*Spodoptera frugiperda*), a polyphagous insect pest, is a major threat to food production, rapidly spreading through all the tropical areas in the world. Resistance has developed to the control protocols used so far (pyrethroids, organophosphorus, and genetically modified plants), and alternative strategies must be found. The bioactivity in essential oils is usually associated with the major constituents, but synergistic interactions among the constituents (even minor ones) can improve the levels of activity considerably. Herein, we tested the insecticidal activity of several constituents of the essential oil from *Piper aduncum*, an Amazonian Piperaceae, both separately and as binary mixtures, through their application on the dorsal side of the larva pronotum. Dillapiole proved to be, isolated, the most active compound in this oil (LD_50_ = 0.35 ppm). In binary mixtures, a strong synergistic effect was observed for the pairs of dillapiole with β-caryophyllene (LD_50_ = 0.03 ppm), methyl eugenol (LD_50_ = 0.05 ppm), and α-humulene (LD_50_ = 0.05 ppm). In some cases, however, antagonism was recorded, as for dillapiole + β-pinene (LD_50_ = 0.44 ppm). The use of binary mixtures of essential oil constituents as low-environmental-toxicity insecticides allows a fine tuning of the insecticidal activity, and the exploitation of synergy effects.

## 1. Introduction

A widespread use of synthetic insecticides for decades has led to their environmental accumulation, and their contamination of soils, groundwater, crops, livestock, food, milk and, at the end of the chain, humans [1,2]. Natural insecticides, mainly from plants, present several advantages: the compounds have shorter half-lives, and are biodegradable and, when they are used in bulk or as multicomponent formulations, insect resistance takes longer to develop, in comparison with synthetic analogues [1]. 

Essential oils are defined as products obtained from natural raw materials of plant origin via physical processes only, such as steam and dry distillation or, in citrus fruits, the pressing of the epicarp [3]. Their composition includes mainly mono- and sesquiterpenes, as well as arylpropanoids.

The use of essential oils as low-risk insecticides, and in organic certified crops, has increased in the last decade [4]. The insecticidal effects are related to repellency or a growing inhibition against phytophagous and, with very few exceptions, their toxicity to mammals is low, as is their environmental persistency [5].

Bioactivity in essential oils is usually associated with a few major compounds. However, synergic interactions among their constituents can result in higher levels of activity, in comparison with the use of isolated major constituents [6,7]. Binary mixtures, for example, presented a higher activity, compared to single compounds [8,9].

The combination of compounds isolated from essential oils can produce additive, synergistic, or antagonistic activities [6]. The identification of synergies may lead to the formulation of commercial insecticides based on standardized mixtures, with a higher efficacy, and lower concentrations required to reach satisfactory levels of insect control [10,11].

Using binary mixtures, Pavela evaluated the interactions among 30 compounds commonly found in essential oils with a proven acute toxicity against the larvae of the cotton leafworm, *Spodoptera littoralis* (Boisduval, 1833) (Lepidoptera: Noctuidae) [11]. After 435 possible combinations were tested, it was observed that only 6 out of the 30 compounds, namely γ-terpinene, limonene, camphor, *p*-cymene, (*E*)-anethole, and borneol, presented significative synergistic interactions in binary combinations, with more than 20 different compounds [11]. Lately, such combinations have become referential in the production of more than 40 different insecticidal formulations based on essential oils that are currently commercialized in North America, Europe, and Asia [12].

*Spodoptera frugiperda* (J.E. Smith, 1797) (Lepidoptera: Noctuidae), commonly known as fall armyworm, is a polyphagous insect pest native to the tropical and subtropical Americas [13]. Although *S. frugiperda* feeds on more than 350 plant species, it has a preference for maize (*Zea mays* L.) [13,14]. Pesticides, pyrethroids, and organophosphorus derivatives, mainly, as well as genetic modified plants expressing *Bacillus thuringiensis* (*Bt*) toxins, have been used as control strategies, but resistance to both has developed [15,16,17]. It is a highly migratory pest and, since its first outbreak in sub-Saharan Africa in 2016, it has already infested several Asian countries and, more recently, reached Australia [14].

The family Piperaceae is comprised of five genera. *Piper* L. is the largest, with circa 2400 species, and a pantropical and subtropical distribution [18]. In Brazil, there are currently 299 species accepted, with 194 of them endemic [19]. *Piper aduncum* L. is a shrub occurring from north to south in virtually all Brazilian biomes [19].

Dillapiole is, frequently, the major constituent in the essential oil from *P. aduncum*, ranging from 50 to 90% of the oil composition, particularly in samples from the Amazon area [20]. Arylpropanoids are recurrently found as major compounds is essential oils from Amazonian Piperaceae [20], many of them containing a methylenedioxyphenyl group. They can interfere with, and inhibit, the main enzyme classes in insects, such as cytochrome P450 dependent monooxygenases, esterases, and glutathione *S*-transferase [21].

This interference could explain the insecticidal effect of the essential oil of *P. aduncum* when applied to the control of several arthropods [22]. Minor components present in the oil also lead to enzymatic inhibition when tested as isolates. Such is the case with limonene (P450 monooxygenase and acetylcholinesterase), myristicin (acetylcholinesterase, carboxylesterases, glutathione *S*-transferase), α-pinene (P450 monooxygenase), β-pinene and *p*-cymene (esterases), and caryophyllene oxide (acetylcholinesterase). Another positive point regarding the use of the essential oil from *P. aduncum* is its shelf-life: it has showed itself to be stable under different storage conditions for at least 4 years [23].

Most of the commercial formulations involving essential oils and their derivatives are based on terpenoids, while combinations among arylpropanoids, and of arylpropanoids and terpenoids, have not been tested [24].

Herein, the occurrence of synergistic interactions in binary combinations of dillapiole with terpenoids and arylpropanoids originally present in the essential oil from *P. aduncum*, regarding the insecticidal activity against *S. frugiperda*, was investigated, for the development of more efficient bioinsecticides.

## 2. Results

Previous analyses of the essential oils from *P. aduncum*, and their rectified fractions, as well as changes during a 4-year shelf-life study, have been published elsewhere [23]. Gas chromatography was used here for the quality control of the compounds (dillapiole, safrole, and asaricin) isolated via fractional distillation. See the experimental section for details.

### 2.1. Acute Toxicity

Seventeen compounds originally present in the essential oil of *P. aduncum* compounds were tested, one by one, against third instar larvae of *S. frugiperda*. Dillapiole was the one with the lower lethal dose (LD_50_ = 0.35 ppm) and, therefore, the most toxic (Table 1 and Table S1, Supplementary Material). It was followed by myristicin (0.62 ppm) and asaricin (0.95 ppm). Arylpropanoid compounds were shown to be the most active, together with tetradecanol.

Oxygenated monoterpenes (linalool and 1,8-cineole) presented an intermediary toxicity (LD_50_ of 2.71 and 3.91, respectively). All three sesquiterpenes tested—β-caryophyllene, α-humulene, and aromadendrene—gave similar LD_50_ values (6.24 ppm, 4.94 ppm and 4.24 ppm, respectively). At the other end of the scale, monoterpenes were the least active, with β-ocimene presenting the highest LD_50_ (12.81 ppm).

**Figure 1:**
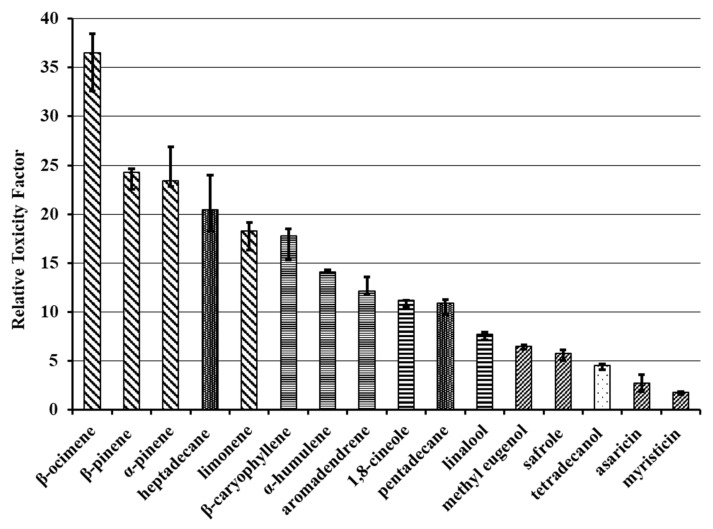
The toxicity of dillapiole relative to the other tested compounds against the third-instar larvae of *S. frugiperda*. The bars represent the confidence interval with 95% of probability, indicating no significant difference between treatments when they are superimposed.

The relative toxicity factor (RTF, Figure 1) was calculated as the ratio between the LD_50_ of each compound tested and that of dillapiole, as expressed in Equation (1):

RTF = LD_50_ (compound)/LD_50_ (dillapiole) (1)

The RTF value is used to express how a compound (in this case, dillapiole) is more active than the other compounds tested in the same study. For myristicin and asaricin, both structurally related to dillapiole (for the structural formulae, see Figure S1 in the Supplementary Material), low RTF values were recorded (1.8 and 2.7, respectively), indicating a similar toxicity (Figure 1). Safrole and methyl eugenol, also arylpropanoid compounds, although with increasing structural dissimilarity towards dillapiole, showed higher RTF values (5.8 and 6.5, respectively). The highest RTF (36.6) was observed for β-ocimene, which highlights the low toxicity of this substance for *S. frugiperda*, in comparison with dillapiole.

### 2.2. Acute Effects of Binary Mixtures

A series of binary mixtures were prepared in a 1:1 proportion of a sublethal dose of dillapiole (LD_40_) and of the second compound (LD_25_). These mixtures were tested, and their LD_50_ values are presented in Table 2. The percentages of each compound in the mixture are given in Table S2 (Supplementary Material).

All mixtures, except those with α-pinene and β-pinene, led to an LD_50_ value lower than that of pure dillapiole. The best association was observed for the pair comprising dillapiole and β-caryophyllene, with an LD_50_ value of 0.03 ppm.

The first observation that can be made based on the data in Table 2 is that the LD_50_ values for the mixtures cannot be predicted, or based only on the chemical class of the compounds added to dillapiole, but are structure-dependent. For example, the lowest LD_50_ values were observed for the combinations of dillapiole with β-caryophyllene, a sesquiterpene, and with methyl eugenol, an arylpropanoid derivative.

The synergy factor (SF) of a mixture of dillapiole and a compound *i* was calculated as the ration between the LD_50_ of pure dillapiole and the LD_50_ of the mixture of dillapiole and the compound *i*.

SF*i* = LD_50_ (pure dillapiole)/LD_50_ (mixture dillapiole + compound *i*) (2)

## 3. Discussion

### 3.1. Acute Toxicity

As can be seen both in Table 1 and Figure 1, the activity varied according to the chemical class of the compounds, which can be expected if one considers that the same mechanism of action is involved. In such cases, structurally-related compounds, acting through the same pathway, produce similar results.

For the substances tested against *S. frugiperda* larvae, arylpropanoids were the most active, followed, in a decreasing order of activity, by oxygenated monoterpenoids, sesquiterpenes, and monoterpene hydrocarbons, as the less active ones. The exception to this rule comprised the linear hydrocarbons: pentadecane (RTF = 10.9) was much more active than heptadecane (RTF = 20.5).

No significative difference was observed for the activities of α-pinene, β-pinene, heptadecane, and limonene. The low activities of α-pinene and β-pinene against *S. frugiperda* (82.1 ppm and 85.2 ppm, respectively) calculated here are in agreement with data published by Pavela, who found LD_50_ values of 87.0 ppm and 85.0 ppm, respectively [11].

No information was found in the literature regarding the insecticidal effect of heptadecane, which showed a low toxicity in comparison with dillapiole (RTF = 20.5, Figure 1).

### 3.2. Acute Effects of Binary Mixtures

There are still few publications regarding comparative binary combinations of essential oils constituents. On the other hand, there is a large amount of information on the use of predominantly terpenic (whole) essential oils as insecticides [25]. Data on synergic interactions among binary combinations of terpenoids and arylpropanoids are still rare.

Terpenoids owe their toxicity against insects predominantly to neurotoxic and growth regulation effects [26,27], after penetration through the cuticula (contact toxicity), digestive system (feed inhibition), or respiratory system (fumigant activity) [28], while arylpropanoids affect the metabolic enzymes of the insets [29].

Octopamine, a biogenic amine, an important neurotransmitter, neuromodulator, and neurohormone in invertebrates, with a biological function analogue to norepinephrine invertebrates, is another target for insecticidal activity among essential oils constituents [30].

A property to be taken into consideration in synergy evaluation is the lipophilicity of the compounds, which plays a fundamental role in the modulation of insecticidal activity. The association among lipophilic compounds, together with protein and/or enzyme inactivation, can provide a reasonable explanation for the perceived effect [31]. This was confirmed in a chemometric study, in which the activity of terpenoids and arylpropanoids was correlated via independent variables to a hydrophobic character [32].

**Figure 2:**
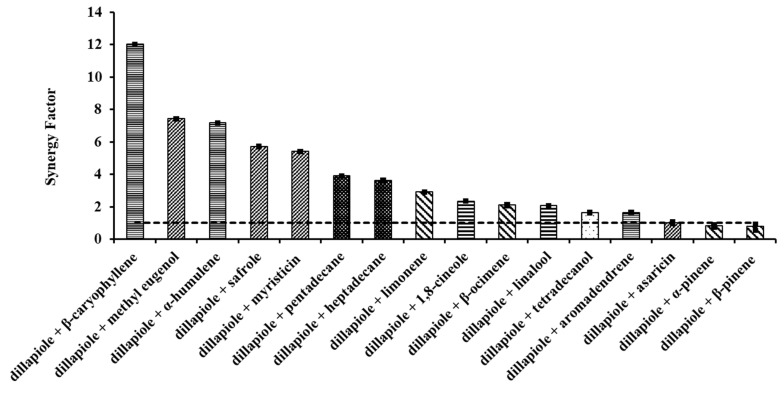
Synergistic factors (SFs) of dillapiole in binary mixtures against *S. frugiperda* larvae. The bars represent the confidence intervals with 95% of probability, indicating no significant difference between treatments when they are superimposed. The dotted line as SF = 1 defines the effect of the combinations as synergic (SF > 1), additive (SF = 1), and antagonistic (SF < 1).

Considering the compounds tested, only two produced a slightly antagonistic effect when combined with dillapiole: α-pinene and β-pinene, with LD_50_ values of 0.42 ppm and 0.44 ppm, respectively (Table 2), and the same synergy factor of 0.8 (Figure 2).

According to published data, both compounds inhibit several cytochrome-P450-dependent monooxygenases and esterases and, for α-pinene, there is also evidence of glutathione S-transferase inhibition [33,34,35]. There is also evidence that α-pinene causes the inhibition of octopamine and γ-aminobutyric acid receptors [36]. In other studies, the total removal of α-pinene from a terpenoid mixture did not significantly reduce the synergic response [37,38]. Although isolated, and less toxic than dillapiole under the test conditions (Table 1), still, an addictive effect could be expected. However, this effect was not verified.

Unpredictable and/or possible conflicting results between one composition and another make the evaluation of each combination a necessity. A better understanding of the divergent points along the detoxification biochemical pathway could clarity the ambiguous effects observed [24].

As can be inferred from the literature, straightforward rules do not apply. In this study, the LD_50_ values of the mixtures (Table 2) and their SFs (Figure 2) could not be predicted, or based only on the chemical class of the compounds added to dillapiole, and were shown to be individually structure-dependent.

For example, the best synergic effects were observed for the association between dillapiole and β-caryophyllene, a sesquiterpene, with an LD_50_ of 0.03 ppm, while for the combination with aromadendrene, the toxicity was seven times lower (LD_50_ = 0.21 ppm). This was also observed in the association of dillapiole with α-humulene, and with methyl eugenol, which led to the same toxicity (LD_50_ = 0.05 ppm). The former is a macrocyclic sesquiterpene, and the latter an oxygenated arylpropanoid. Therefore, the synergism must be tested for every combination.

Sesquiterpenes are lipophilic compounds and, therefore, could act to facilitate the passage of dillapiole through the larva tegument, by disrupting biomembranes [39]. For aromadendrene, specifically, a stronger synergism has been observed in other systems tested, such as *Tetranychus urticae* (Koch, 1836) (Acari: Tetranychidade) [38] but, again, synergism is structure-dependent and target-dependent.

No significative difference was observed among the SF values for aromadendrene, a sesquiterpene, and tetradecanol, a fatty alcohol (both with SF = 1.6). Although it has a similar molecular mass, aromadendrene is a tricyclic sesquiterpene, structurally very restrained, with no oxygen atoms present. Tetradecanol is an oxygenated linear compound, and can assume quite different conformations in response to its chemical environment. Nevertheless, both compounds are lipophilic, which could suggest a similar mechanism of action and, therefore, the same synergy factor.

Ocimene is an important volatile in allelopathic processes [40]. It showed a low toxicity in comparison to that of dillapiole, and only a small synergic effect when associated with it (SF = 2.10). The same valor was recorded for linalool (Figure 2). Linalool is considered to be an inhibitor of P450 monooxygenases and esterases [41,42,43]. It exhibited an intermediary toxicity compared to dillapiole. The binary combination of both, however, led only to a small synergic effect (SF = 2.1). This was not anticipated; once, the synergic association of linalool with piperonyl butoxide, for example, increased the toxicity 27 times when tested against third-instar larvae of *Aedes* (*Stegomyia*) *aegypti* (Linnaeus, 1762) [44].

Dillapiole, asaricin, myristicin, and safrole are arylpropanoids, all containing the basic 5-(2-propenyl)-1,3-benzodioxole structure. Asaricin and myristicin are positional isomers, with one methoxy substituent in the aromatic ring. Dillapiole has two (Figure S1). For asaricin, an additive effect to dillapiole was observed (SF = 1.0), which could be associated with its esterase inhibition properties [45]. Myristicin, on the other hand, showed a synergic interaction with dillapiole (SF = 5.4), quite close to that of safrole, with SFs = 5.7.

The use of binary mixtures of essential oil constituents as low environmental toxicity insecticides has the advantage of allowing a fine tuning on the insecticidal activity, and the exploitation of synergy effects. Through the combination of different mechanisms of action in a single product, resistance development is reduced or, at least, retarded.

Some of the formulations tested in vitro are currently under trial in the field.

## 4. Materials and Methods

### 4.1. Plant Material

Samples of *P. aduncum* were obtained from cultivated plants at the Active Germplasm Bank of Embrapa Acre (10°15′57′′ S, 67°42′17′′ W), voucher 8817. The species taxonomy was determined by Dr. Elsie F. Guimarães from the Botanical Garden of Rio de Janeiro. The authorization for access to Brazilian biodiversity was acknowledged via the IBAMA permits 02001.050950/2011-61 for scientific research, and 02000.000460/2013-96 for bioprospection.

### 4.2. Essential Oil Distillation and Rectification

The essential oil of P. aduncum was steam-distilled in a 200 L still (Ercitec, Bauru SP, Brazil), in batches of 150 kg, with cohobation of the condensed waters. The oil was then rectified via fractional distillation in a 30 L Ercitec still, at atmospheric pressure.

### 4.3. Chemicals

The chemical standards used, namely ocimene (94%); limonene (96%) and α-humulene (96%); myristicin and aromadendrene (97%); α-pinene and β-pinene (98%); linalool, 1,8-cineole, β-caryophyllene, eugenol, pentadecane, heptadecane and tetradecanol (99.0%) were all purchased from Sigma-Aldrich (Saint Louis, MO, USA).

Dillapiole, asaricin, and safrole, all with a purity higher than 99.7%, were obtained via fractional distillation, as described in Section 4.2.

### 4.4. Analyses of the Essential Oil and Distilled Fractions

A 1% solution of the distillate samples in dichloromethane was prepared, and 1 μL was injected, in split mode (1:50), into an Agilent 7890A gas chromatograph (GC) fitted with a 7693B automatic sampler and a flame ionization detector (FID). The FID was operated at 280 °C. The separation of the components was obtained via a HP-5MS fused silica capillary column (5%-phenyl-95%-methyl-silicone, 30 m × 0.25 mm × 0.25 μm). Hydrogen was used as a carrier gas (1.5 mL min^−1^). The oven temperature was programed from 60 to 240 °C at 3 °C min^−1^. The quantification was based on the area (area%) from the signal of the FID normalized with an internal standard (methyl octanoate). All analyses were made in triplicate. Analyses via mass spectrometry (GC-MS) were performed on an Agilent 5975C mass selective detector coupled to an Agilent 7890A gas chromatograph, with the same column, temperatures, and injection conditions as above. Helium was used as a carrier gas at 1 mL min^−1^. The mass detector was operated in electron ionization mode (70 eV), at 3.15 scans sec^−1^, with a mass range from 40 to 450 u. The transfer line was kept at 240 °C, the ion source at 230 °C, and the analyzer at 150 °C.

### 4.5. Acute Toxicity of Compounds

All toxicological assays were performed at the Laboratory of Entomology of Embrapa Acre, using third-instar larvae of *S. frugiperda*, bred with an artificial diet [46]. Authorization for larva reproduction was obtained (license SISBIO 13464-2). Each compound was tested in isolation to evaluate the insecticidal effect via topical contact, using the protocol of Estrela et al. [47].

To determine the experimental parameters for the bioassays, preliminary tests were conducted, using a completely randomized design, with four replicates per treatment [48]. Each replicate consisted of a Petri dish with 10 larvae. For all compounds tested, using a micro syringe, 1 µL was applied to the dorsal side of the larva pronotum [49]. The larvae were then kept for 24 h, without feeding. After establishing the overall response range for the nearly-zero to nearly-100% concentrations of larva mortality, narrower response ranges were determined [50]. Seven concentrations were defined for topical contact bioassays.

In all the experiments, acetone was used as a solvent, and as a negative control. After the application of the compounds to be tested, the Petri dishes were kept in an incubator at a temperature of 25 ± 2 °C, 70 ± 5% relative humidity, and a photophase of 12 h. After 24 h, the larval mortality was evaluated, and corrected according to the natural one, using Abbott’s mathematical adjustment [51].

The lethal doses at 25% and 50% of mortality (LD_25_ and LD_50_) were determined by the concentration x mortality curves and confidence intervals (95% CI) using Probit analysis and the SAS program. The Probit analyses followed the method described by Finney [50]. To test the goodness-of-fit, Pearson’s chi-square test (χ2) was used, with a significance level of 5%.

### 4.6. Acute Effects of Binary Mixtures with Dillapiole

Initially, the relative toxicity factor (RTF) was calculated according to the ratio between the LD_50_ of each compound tested and the LD_50_ of dillapiole. A synergistic interaction was considered to occur when the combination of the sublethal doses of two compounds resulted in a significant higher lethality relative to the individual effect of each pure compound [52].

Using the RTF concept, a sublethal dose (LD_40_) of dillapiole was combined with a sublethal dose (LD_25_) of each of the selected compounds, as binary combinations in a 1:1 proportion. Following the procedure described above, and using Probit analysis, new LD_50_ values were obtained for each combination tested.

To evaluate the synergic effect for each combination, a synergy factor (SF) was calculated according to the ratio between the LD_50_ of pure dillapiole and that of the binary combination [53].

The synergic effect of each combination was considered significant if no overlapping in the confidence intervals (at 95%) of the synergy factor occurred for the combinations evaluated [54]. SF values above 1.0 indicated a synergistic effect; those equal to 1.0, an additive effect; and those below 1.0, an antagonist response, in relation to each compound combined with dillapiole [55].

## 5. Conclusions

The insecticidal activity of 16 constituents from the essential oil of *Piper aduncum*, isolated or as binary mixtures, was tested with regard to acute toxicity in third-instar larvae of *Spodoptera frugiperda*. Dillapiole was the most active as a pure compound (LD_50_ = 0.35 ppm). Among the binary mixtures tested, a strong synergistic effect was observed for the combination of dillapiole with β-caryophyllene (LD_50_ = 0.03 ppm), methyl eugenol (LD_50_ = 0.05 ppm), and α-humulene (LD_50_ = 0.05 ppm). On the other hand, a slight antagonistic effect was registered for the combination of dillapiole with β-pinene.

The use of essential oils, and mixtures of their constituents, as natural insecticides has the advantages of a low environmental toxicity, and the possibility of adjustment of the insecticidal activity, to explore synergistic interactions, and minimize or delay the development of insect resistance.

The essential oil *P. aduncum* has been studied as a natural insecticide, either pure or in combination with other oils. Few studies have been published, however, concerning the effect of binary or more complex combinations of oil constituents, such as terpenoids and arylpropanoids. The results presented here offer a contribution to the knowledge on the interactions and effects of such combinations.

## Figures and Tables

**Table 1:** Lethal doses (LD_50_) for individual tested compounds to *S. frugiperda* third instar larvae.

Compounds	LD_50_ (ppm)	DF	Prob. χ^2^	Pearson’s χ^2^	R^2^	Angular Coefficient ± SEM
dillapiole	0.35	22	0.7040	18.0332	0.72	1.17 ± 0.15
myristicin	0.62	18	0.8764	11.4053	0.88	1.84 ± 0.16
asaricin	0.95	26	0.3946	24.4778	0.66	0.33 ± 0.05
tetradecanol	1.59	22	0.4554	22.0761	0.79	0.93 ± 0.10
safrole	2.02	26	0.5895	23.7626	0.81	2.05 ± 0.20
methyl eugenol	2.28	22	0.3867	23.2679	0.82	1.03 ± 0.10
linalool	2.71	38	0.1225	48.1414	0.85	0.90 ± 0.06
pentadecane	3.81	18	0.7978	12.8948	0.89	1.85 ± 0.15
1,8-cineole	3.91	34	0.9993	1.6763	0.88	0.94 ± 0.06
aromadendrene	4.24	18	0.2576	21.4431	0.75	0.64 ± 0.09
α-humulene	4.94	20	0.1034	28.2604	0.75	0.83 ± 0.11
β-caryophyllene	6.24	16	0.6323	13.5492	0.80	0.49 ± 0.06
limonene	6.41	46	0.9988	16.6017	0.86	1.33 ± 0.08
heptadecane	7.17	13	0.7230	9.6409	0.73	0.29 ± 0.05
α-pinene	8.21	17	0.4060	17.7305	0.90	0.68 ± 0.05
β-pinene	8.52	18	0.0661	27.7428	0.66	0.90 ± 0.15
β-ocimene	12.81	22	0.9044	13.9184	0.85	1.61 ± 0.14

LD_50_ = lethal doses causing 50% of mortality of insects; DF = degrees of freedom; Prob. = probability chi-square; R^2^ = coefficient of determination; SEM = standard error.

**Table 2:** Lethal dose (LD_50_) values for the binary mixtures tested against *S. frugiperda* third-instar larvae.

Binary Mixtures	LD_50_ (ppm)	DF	Prob. χ^2^	Pearson’s χ^2^	R^2^	Angular Coefficient ± SEM
dillapiole + β-caryophyllene	0.03	28	0.8542	20.2750	0.85	0.26 ± 0.02
dillapiole + methyl eugenol	0.05	22	1.6690	28.2693	0.85	0.62 ± 0.05
dillapiole + α-humulene	0.05	22	0.8690	14.8408	0.89	0.52 ± 0.04
dillapiole + safrole	0.06	26	0.9123	16.8910	0.74	0.27 ± 0.03
dillapiole + myristicin	0.05	22	0.9887	9.7158	0.87	0.50 ± 0.04
dillapiole + pentadecane	0.09	26	0.1261	34.3629	0.72	0.58 ± 0.07
dillapiole + heptadecane	0.10	22	0.6245	19.3366	0.86	1.08 ± 0.09
dillapiole + limonene	0.12	22	0.8088	16.1412	0.83	0.90 ± 0.09
dillapiole + 1,8 cineole	0.15	22	0.9257	13.2597	0.83	0.95 ± 0.09
dillapiole + β-ocimene	0.17	22	0.9066	13.8542	0.84	0.66 ± 0.06
dillapiole + linalool	0.17	30	0.1910	36.5375	0.71	0.83 ± 0.10
dillapiole + tetradecanol	0.21	14	0.1258	20.1389	0.71	1.17 ± 0.20
dillapiole + aromadendrene	0.21	14	0.6326	11.6721	0.81	2.14 ± 0.28
dillapiole + asaricin	0.34	17	0.7140	13.3269	0.88	0.69 ± 0.06
**pure dillapiole**	**0.35**	**22**	**0.7040**	**18.0332**	**0.72**	**1.17 ± 0.1**
dillapiole + α-pinene	0.42	20	0.3115	22.5492	0.80	0.58 ± 0.06
dillapiole + β-pinene	0.44	17	0.1183	24.0415	0.69	0.36 ± 0.06

LD_50_ = lethal dose causing 50% mortality in insects; DF = degrees of freedom; Prob. = probability chi-square; R^2^ = coefficient of determination; SEM = standard error.

## Supplementary Materials

The following supporting information can be downloaded at: https://www.mdpi.com/article/10.3390/plants12173042/s1, Figure S1: Structural formula of the compounds tested; Table S1: The lethal dose (LD_50_) with confidence intervals for each individual tested compound to *S. frugiperda* third-instar larvae; Table S2: Percentages of each constituent in the mixture and lethal dose (LD_50_) with confidence intervals for the binary mixtures tested against *S. frugiperda* third-instar larvae.
