# Supplementary material for: Synergism in Two-Component Insecticides with Dillapiole against Fall Armyworm
Source: Plants (Basel). 2023 Aug 24;12(17):3042. doi: 10.3390/plants12173042 (PMC10489978; doi:10.3390/plants12173042)
Supplement: Supplementary file 1 [file plants-12-03042-s001.zip › plants-2554530-supplementary.pdf]

Supplementary material

Synergism in two-component insecticides with dillapiole against fall armyworm

Murilo Fazolin [1,*], Humberto R. Bizzo [2,*], André F. M. Monteiro [1], Maria E. C. Lima [3], Natalia S. Maisforte [4] and Paola E. Gama [2]

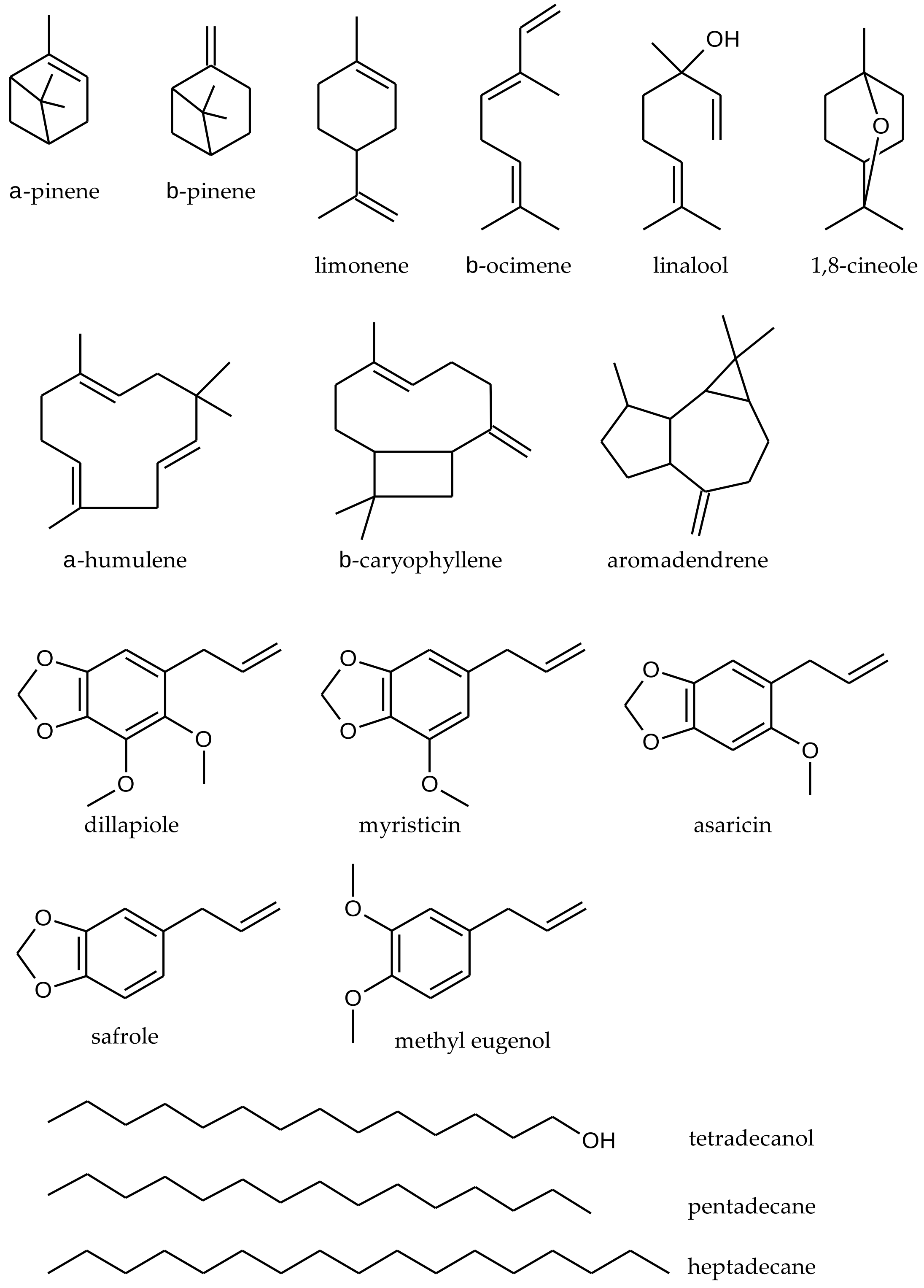

Figure S1. Structural formula of the compounds tested.

Table S1. Lethal doses (LD_{50}) with confidence intervals for individual tested compounds to *S. frugiperda* third instar larvae.

Compounds	LD_{50} (95% CI) (ppm)	DF	Prob. χ^2	Pearson χ^2	R^2	Angular Coefficient ± SEM
dillapiole	0.35 (0.29 - 0.40)	22	0.7040	18.0332	0.72	1.17 ± 0.15
myristicin	0.62 (0.58 - 0.66)	18	0.8764	11.4053	0.88	1.84 ± 0.16
asaricin	0.95 (0.55 - 1.41)	26	0.3946	24.4778	0.66	0.33 ± 0.05
tetradecanol	1.59 (1.44 - 1.74)	22	0.4554	22.0761	0.79	0.93 ± 0.10
safrole	2.02 (1.89 - 2.15)	26	0.5895	23.7626	0.81	2.05 ± 0.20
methyl eugenol	2.28 (2.00 - 2.68)	22	0.3867	23.2679	0.82	1.03 ± 0.10
linalool	2.71 (2.43 - 2.98)	38	0.1225	48.1414	0.85	0.90 ± 0.06
pentadecane	3.81 (3.50 - 4.15)	18	0.7978	12.8948	0.89	1.85 ± 0.15
1,8-cineole	3.91 (3.44 - 4.39)	34	0.9993	1.6763	0.88	0.94 ± 0.06
aromadendrene	4.24 (3.45 - 5.39)	18	0.2576	21.4431	0.75	0.64 ± 0.09
α-humulene	4.94 (4.12 - 5.67)	20	0.1034	28.2604	0.75	0.83 ± 0.11
β-caryophyllene	6.24 (4.49 - 8.93)	16	0.6323	13.5492	0.80	0.49 ± 0.06
limonene	6.41 (5.95 - 6.92)	46	0.9988	16.6017	0.86	1.33 ± 0.08
heptadecane	7.17 (3.88 - 17.45)	13	0.7230	9.6409	0.73	0.29 ± 0.05
α-pinene	8.21 (6.69 - 10.65)	17	0.4060	17.7305	0.90	0.68 ± 0.05
β-pinene	8.52 (6.61 - 9.76)	18	0.0661	27.7428	0.66	0.90 ± 0.15
β-ocimene	12.81 (11.84 - 13.72)	22	0.9044	13.9184	0.85	1.61 ± 0.14

LD_{50} = lethal doses causing 50% of mortality of insects; 95% CI = confidence interval with 95% of probability; DF = degrees of freedom; Prob. = probability chi-square; R^2 = coefficient of determination; SEM = standard error.

Table S2. Lethal doses (LD_{50}) with confidence intervals for binary mixtures tested to *S. frugiperda* third instar larvae.

Binary mixtures	LD_{50} (95% CI) (ppm)	DF	Prob. χ^2	Pearson χ^2	R^2	Angular Coefficient ± SEM
dillapiole (0,3%) + β-caryophyllene (2,1%)	0.03 (0.02 - 0.05)	28	0.8542	20.2750	0.85	0.26 ± 0.02
Dillapiole (0,3%) + methyl eugenol (0,2%)	0.05 (0.04 - 0.06)	22	1.6690	28.2693	0.85	0.62 ± 0.05
dillapiole (0,3%) + α-humulene (3,1%)	0.05 (0.04 - 0.06)	22	0.8690	14.8408	0.89	0.52 ± 0.04
dillapiole (0,3%) + safrole (1,6%)	0.06 (0.04 - 0.10)	26	0.9123	16.8910	0.74	0.27 ± 0.03
dillapiole (0,3%) + myristicin (0,5%)	0.06 (0.05 - 0.09)	22	0.9887	9.7158	0.87	0.50 ± 0.04
dillapiole (0,3%) + pentadecane (2,9%)	0.09 (0.07 - 0.11)	26	0.1261	34.3629	0.72	0.58 ± 0.07
dillapiole (0,3%) + heptadecane (1,1%)	0.10 (0.08 - 0.11)	22	0.6245	19.3366	0.86	1.08 ± 0.09
dillapiole (0,3%) + limonene (0,6 %)	0.12 (0.10 - 0.14)	22	0.8088	16.1412	0.83	0.90 ± 0.09
dillapiole (0,3%) + 1,8 cineole (2,2%)	0.15 (0.13 - 0.17)	22	0.9257	13.2597	0.83	0.95 ± 0.09
dillapiole (0,3%) + β-ocimene (10,3%)	0.17 (0.12 - 0.21)	22	0.9066	13.8542	0.84	0.66 ± 0.06
dillapiole (0,3%) + linalool (1,8%)	0.17 (0.15 - 0.20)	30	0.1910	36.5375	0.71	0.83 ± 0.10
dillapiole (0,3%) + tetradecanol (1,2%)	0.21 (0.19 - 0.25)	14	0.1258	20.1389	0.71	1.17 ± 0.20
dillapiole (0,3%) + aromadendrene (2,2%)	0.21 (0.20 - 0.23)	14	0.6326	11.6721	0.81	2.14 ± 0.28
dillapiole (0,3%) + asaricin (0,2%)	0.34 (0.28 - 0.42)	17	0.7140	13.3269	0.88	0.69 ± 0.06
**pure dillapiole**	**0.35 (0.29 - 0.40)**	**22**	**0.7040**	**18.0332**	**0.72**	**1.17 ± 0.15**
dillapiole (0,3%) + α-pinene (4,7%)	0.42 (0.34 - 0.53)	20	0.3115	22.5492	0.80	0.58 ± 0.06
dillapiole (0,3%) + β-pinene (6,5%)	0.44 (0.30 - 0.46)	17	0.1183	24.0415	0.69	0.36 ± 0.06

LD_{50} = lethal doses causing 50% of mortality of insects; 95% CI = confidence interval with 95% of probability; DF = degrees of freedom; Prob. = probability chi-square; R^2 = coefficient of determination; SEM = standard error.
